# Development of a colloidal gold immunochromatographic strip for the rapid detection of fowl adenovirus serotype 4 using prepared penton protein-specific monoclonal antibodies

**DOI:** 10.3389/fvets.2026.1758143

**Published:** 2026-03-05

**Authors:** Sisi Luo, Bingyi Yang, Jiaoling Huang, You Wei, Zhixun Xie, Xiaofeng Li, Aiqiong Wu, Zhihua Ruan, Sheng Wang, Yanfang Zhang, Meng Li, Liji Xie, Ming Yan, Weiwei Wang, Ping Wei

**Affiliations:** 1 Institute for Poultry Science and Health, College of Animal Science and Technology, Guangxi University, Nanning, Guangxi, China; 2 Key Laboratory of China (Guangxi)-ASEAN Cross-Border Animal Disease Prevention and Control, Ministry of Agriculture and Rural Affairs, Guangxi Key Laboratory of Veterinary Biotechnology, Guangxi Veterinary Research Institute, Nanning, Guangxi, China

**Keywords:** colloidal gold immunochromatography, fowl adenovirus serotype 4, monoclonal antibody, penton protein, rapid diagnosis

## Abstract

Fowl adenovirus serotype 4 (FAdV-4) is the primary pathogen responsible for hydropericardium-hepatitis syndrome (HHS) and is associated with high mortality rates (20–80%) in 3–6-week-old chickens. This study aimed to develop a rapid and specific method for detecting FAdV-4. Two monoclonal antibodies (mAbs 6B3 and 8G11) against the FAdV-4 penton protein were successfully generated using hybridoma technology, both of which exhibited high titers (1:100,000) and strong serotype specificity. Specificity analysis confirmed that these mAbs recognized 12 FAdV-4 isolates from diverse origins without cross-reactivity to other FAdV serotypes or common avian pathogens. On this basis, a colloidal gold immunochromatographic assay was developed; systematic optimization of its key parameters yielded an optimal labeling pH of 8.3, an antibody labeling concentration of 7.2 μg/mL, and optimal coating concentrations for the test line (T-line) and control line (C-line) of 2.5 mg/mL and 1.5 mg/mL, respectively. Performance evaluation of the method demonstrated that it achieved a detection sensitivity of 7.81 × 10^4^ TCID₅₀/0.1 mL with a detection time of 15 min. Clinical sample validation revealed a positive concordance rate of 91.6%, a negative concordance rate of 100%, and an overall concordance rate of 98.6% relative to those of the ELISAs. The proposed colloidal gold immunochromatographic method offers advantages such as ease of operability, rapid visualization and high specificity and serves as a practical technical tool for onsite rapid diagnosis and epidemiological surveillance of FAdV-4 infection, thus it has high potential in the prevention and control of HHS.

## 1 Introduction

Hydropericardium hepatitis syndrome (HHS) is an acute, highly contagious infectious disease caused by fowl adenovirus serotype 4 (FAdV-4) (1, 2) and was first reported in Angara Goth, Pakistan, in 1987 (3, 4). Owing to its typical pathological manifestations, including pericardial effusion and hepatic inflammation, it is also known as “Angara disease” (5). With the rapid growth of the poultry industry, HHS has become prevalent in many countries worldwide, resulting in significant economic losses. Epidemiological investigations have indicated that HHS, characterized by an acute onset and a high mortality rate, primarily affects 3–5-week-old chicks (4–6). The hallmark lesions consist of large amounts of clear yellowish fluid or jelly like exudate in the pericardial sac.

FAdV-4 belongs to the genus *Aviadenovirus*. Its genome encodes various structural and nonstructural proteins, among which the hexon, penton and fiber proteins are major components of the viral capsid and play critical roles in viral entry and immune responses (7). Penton is responsible for the internalization of viruses during the infection cycle. Its surface contains type-specific antigenic epitopes, providing a molecular basis for establishing serotype-specific detection methods (8). In recent years, immunofluorescence studies have revealed the expression dynamics and subcellular localization patterns of the penton protein during viral replication, offering important theoretical insights for understanding viral infection mechanisms and developing targeted detection methods (9).

Current diagnostic methods for HHS mainly rely on virus isolation, electron microscopy, agar gel immunodiffusion, serum neutralization tests, ELISAs and PCR (10–19). However, these methods have limitations: virus isolation, serving as the “gold standard,” is time-consuming (typically requiring 5–7 days); electron microscopy is unsuitable for rapid diagnosis; PCR has high equipment and operational requirements; and agar gel immunodiffusion and neutralization tests have shortcomings in terms of sensitivity and specificity. These drawbacks hinder the rapid diagnosis and timely control of HHS.

The colloidal gold immunochromatography assay (CGIA) is a rapid immunoassay technology based on specific antigen–antibody reactions (20, 21). Owing to its advantages of ease of operability, rapid response and easily visualizable results, it has been widely applied for field and rapid laboratory screening (22–24). Its fundamental principle involves immobilizing antibody-labeled colloidal gold particles on a nitrocellulose (NC) membrane, where target analytes in the sample bind to the gold-labeled antibodies via chromatographic action and produce color development, enabling qualitative or semi-quantitative detection of the target (25).

In this study, the FAdV-4 penton protein was successfully expressed using a prokaryotic expression system. After BALB/c mice were immunized, two hybridoma cell lines (6B3 and 8G11) that stably secreted monoclonal antibodies against the penton protein were obtained through cell fusion technology. Immunofluorescence analysis confirmed the specific recognition of the obtained monoclonal antibodies for the FAdV-4 penton protein in infected cells, providing reliable antibody tools for the subsequent development of the proposed detection method. Using the purified monoclonal antibodies(mAbs) as gold-labeled antibodies, detection line (T-line) coating antibodies, and goat anti-mouse IgG as the control line (C-line), a colloidal gold immunochromatographic strip for the rapid detection of FAdV-4 was developed.

## 2 Materials and methods

### 2.1 Experimental materials

The FAdV-4 Guangxi isolates (GX001, GX003–GX005, GX007–GX010, GX012, GX013, GX015, and GX017), as well as H9 subtype avian influenza virus (AIV H9), Newcastle disease virus (NDV), infectious bronchitis virus (IBV), infectious laryngotracheitis virus (ILTV), egg drop syndrome virus (EDSV) and avian Reovirus (ARV) were preserved and provided by the Guangxi Key Laboratory of Veterinary Biotechnology. Six reference strains of FAdV (FAdV-2/5/6/7/11/12) were purchased from the China Institute of Veterinary Drug Control. Female SPF-grade BALB/c mice (6–8 weeks old) were obtained from the Experimental Animal Center of Guangxi Medical University.

Hybridoma cell lines (6B3 and 8G11) that secrete monoclonal antibodies against the FAdV-4 penton protein were previously established and cryopreserved in our laboratory (26). For this study, frozen cells were revived from liquid nitrogen storage.

DMEM (SH30021.01) was purchased from HyClone; fetal bovine serum (10099141) was purchased from Invitrogen; 96-well microplates (FEP101296) were purchased from JET BIOFIL; bovine serum albumin (BSA, A8020) and TMB substrate (T9230) were purchased from Beijing Solarbio Technology; and HRP-labeled goat anti-mouse IgG (5220–0341) and FITC-labeled goat anti-mouse IgG (5230–0427) antibodies were purchased from SeraCare(KPL).

The nitrocellulose membrane (NC membrane, CN140) was a product of Sartorius; 40 nm colloidal gold solution, glass fiber membranes (RB65, SB08), absorbent paper, and polyvinyl chloride (PVC) backing cards were all purchased from Shanghai Jinbio Biotechnology.

### 2.2 Ascites production and antibody purification

Female BALB/c mice (≥8 weeks old) were primed by intraperitoneal injections of 1.0 mL of sterile paraffin oil. After 7 days, 1 × 10^6^ log-phase hybridoma cells (6B3 or 8G11) were injected intraperitoneally. Antibodies in ascites fluid were subjected to twofold serial dilutions for titer determination by indirect ELISA. Ascitic fluid was collected from hybridoma cells 14 days post-inoculation and purified using a Protein A + G Agarose affinity chromatography kit (Beyotime). The concentration of protein antibody in the ascitic fluid was determined with a quantitative ultraviolet spectrophotometer.

### 2.3 Monoclonal antibody characterization

#### 2.3.1 SDS–PAGE analysis

Purified monoclonal antibody samples were mixed with 4 × loading buffer, boiled for 5 min, loaded (20 μL per well), and electrophoresed at a constant voltage (140 V for 40 min). After Coomassie Brilliant Blue staining and destaining, the gels were imaged for analysis.

#### 2.3.2 Dot-ELISA assay

NC membranes were activated by soaking in PBS, spotted with 10 μL of viral samples (including 12 FAdV-4 isolates, FAdV-2/5/6/7/11/12 reference strains and AIV H9, NDV, IBV, ILTV, EDSV and ARV viruses), blocked with 2% BSA-PBS at 37 °C for 1 h, washed with PBST, incubated with appropriately diluted monoclonal antibodies at 37 °C for 1 h, washed again, treated with 1:3000 diluted HRP-labeled goat anti-mouse IgG, and observed after termination of color development.

#### 2.3.3 Indirect immunofluorescence assay

LMH cells were seeded in 12-well plates (containing coverslips), cultured in monolayers, infected with FAdV-4 strains at an MOI of 0.5 and cultured for 96 h. After fixation with 4% paraformaldehyde, permeabilization with 0.2% Triton X-100, and blocking with 2% BSA, the cells were sequentially incubated with mouse anti-penton monoclonal antibodies (6B3 or 8G11) and a FITC-labeled goat anti-mouse IgG secondary antibody for immunostaining. To further determine the subcellular localization of the penton protein, the cells were counterstained with DAPI to visualize the nuclei and imaged by fluorescence microscopy. Uninfected cells served as negative controls.

### 2.4 Development of the colloidal gold Immunochromatographic strip

#### 2.4.1 Preparation of colloidal gold immunochromatographic strip

The sample pad (SB08) was immersed in PBS containing 1% sucrose, 1% BSA, 0.5% Tween-20, and 0.05% ProClin 300 for 30 min, while the conjugate pad (RB65) was treated with an aqueous solution containing 3% sucrose, 2% BSA and 0.05% ProClin 300. After drying, the conjugate pad was uniformly sprayed with gold-labeled antibody(6B3 monoclonal antibody) and further dried for subsequent use.

The nitrocellulose (NC) membrane was cut to a size of 25 × 30 mm. Using a dispenser, the T-line was coated with the monoclonal antibody 8G11 and the C-line was coated with goat anti-mouse IgG. The distance between the T-line and C-line was maintained at 5–8 mm. The coated NC membrane was then dried and stored at 4 °C for later use.

#### 2.4.2 Colloidal gold immunochromatographic strip assembly

A PVC backing card was used as the base. The sample pad, conjugate pad, NC membrane and absorption pad were sequentially overlapped and adhered onto the card with a 2 mm overlap between adjacent components. After assembly, the card was cut into 4 mm-wide strips using a cutting machine. The finished strips were packaged in sealed bags containing desiccant and stored at 4–8 °C. For detection, the sample was simply added to the sample well.

#### 2.4.3 Detection using colloidal gold immunochromatographic strip

##### 2.4.3.1 Sample preparation

The soft tip of the swab was gently inserted into the oropharynx of chicken. The swab was rotated firmly against the inner wall of the oropharynx at least 5 times(for approximately 15 s). The swab (with the sample) was immediately inserted into the extraction tube. Pinch the bottom of the tube and vigorously swirl the swab at least 10 times. After the swab was left in the tube for 3 min, the swabs were squeezed the sides of the tube and removed, and the sample solution was used for detection. Other samples such as cell culture samples and tissue samples need to be processed in the conventional way first, and then centrifuged at 3500 rpm for 3–5 min, after which the supernatant is taken for testing.

##### 2.4.3.2 Testing procedures and result determination

The test strip was removed from its sealed bag and placed horizontally on a clean surface. Using a precision pipette, 100 μL of the prepared sample solution was vertically added to the sample well of the strip. The reaction was allowed to proceed at room temperature. The results could initially be observed 5 min after sample application, with the most stable results obtained at 15 min. To avoid non-specific background interference, all the results must be interpreted within 20 min of sample application. The results were interpreted on the basis of color development at the T-line and C-line: the test was considered positive if both the C-line and T-line displayed distinct red bands, indicating the presence of FAdV-4 antigen in the sample; a negative result was recorded if only the C-line showed a red band with no visible color at the T-line, suggesting that the FAdV-4 antigen was not detected or was below the detection limit; and the test was deemed invalid if no red band appeared at the C-line regardless of T-line development, which could be due to strip failure or improper operation, requiring retesting with a new strip.

#### 2.4.4 Optimization conditions

##### 2.4.4.1 pH optimization for colloidal gold labeling

Nine tubes containing 500 μL of 40 nm colloidal gold solutions were prepared. To each tube, 0–8 μL of 0.1 M K₂CO₃ was added to adjust the pH gradient. Subsequently, 10 μL of 0.5 mg/mL 6B3 monoclonal antibody (final concentration: 10 μg/mL) was added to each tube, followed by 15 min of incubation at room temperature. To assess stability, 100 μL of 10% NaCl solution was added, and the color transition (from red to blue/purple) was observed. The optimal pH (typically pH 8.3) was determined on the basis of the minimal color change, indicating effective antibody conjugation without nanoparticle aggregation.

##### 2.4.4.2 Screening of the optimal antibody labeling concentration

Under the predetermined optimal pH, varying concentrations (0–10 μg/mL) of the 6B3 mAb were added to 500 μL of the colloidal gold solution. After incubation, NaCl challenge tests were performed to evaluate the stability. The minimum antibody concentration that prevented colloidal gold aggregation (indicated by maintenance of a red color) was identified. To ensure robustness, the final working concentration was set at 120% of this threshold.

##### 2.4.4.3 Preparation of the gold-labeled antibody

Under the predetermined optimal pH, the 6B3 mAb with optimal concentration was added to 1 mL of colloidal gold solution. The mixture was incubated with rotation at room temperature for 30 min. Then, 100 μL of 10% BSA was added for blocking. After centrifugation at 2500 rpm for 10 min, the supernatant was discarded. The pellet was resuspended in PBS containing 5% sucrose, 5% trehalose, 1% BSA, 0.5% Tween-20, and 0.05% ProClin 300 and stored at 4 °C.

##### 2.4.4.4 Optimization of the T-line and C-line antibody concentrations

The coating concentrations for the T-line (8G11 monoclonal antibody) and C-line (goat anti-mouse IgG) were systematically optimized within the ranges of 0.5–3.5 mg/mL and 0.5–2.0 mg/mL, respectively. This process involved testing gradient concentrations to balance the signal intensity, background interference and line consistency, ensuring optimal immunochromatographic performance for subsequent evaluations.

### 2.5 Test strip performance evaluation

#### 2.5.1 Specificity test

The test strips were evaluated for cross-reactivity using FAdV-4 isolates (GX001/005/006/007/009), other FAdV serotypes (FAdV-2/5/6/7/11/12) and common avian pathogens (AIV H9, NDV, IBV, ILTV, EDSV and ARV). PBS served as the negative control.

#### 2.5.2 Sensitivity test

A 2-fold serial dilution of the FAdV-4 virus was tested to determine the limit of detection (LOD). The minimum detectable concentration was defined as the last dilution yielding a visible T-line.

#### 2.5.3 Repeatability test

Three samples were tested using test strips from the same batch, each of which was repeated three times, to evaluate intra-batch repeatability. And the three samples were tested using test strips from three different batches, to evaluate inter-batch repeatability.

#### 2.5.4 Stability test

Strips stored at 4 °C in the dark were assessed monthly for 3 months using three positive and three negative samples. The performance remained stable, with no loss of sensitivity or increase in background noise.

#### 2.5.5 Comparative evaluation with ELISA methodology

A total of 152 cloacal and oral swabs of chicken were collected from a Guangxi farm. The test strips were used to simultaneously detect 152 clinical samples with the laboratory’s existing FAdV-4 ELISA method. The agreement rate was calculated.

## 3 Results

### 3.1 Monoclonal antibody preparation and characterization

**Figure 1:**
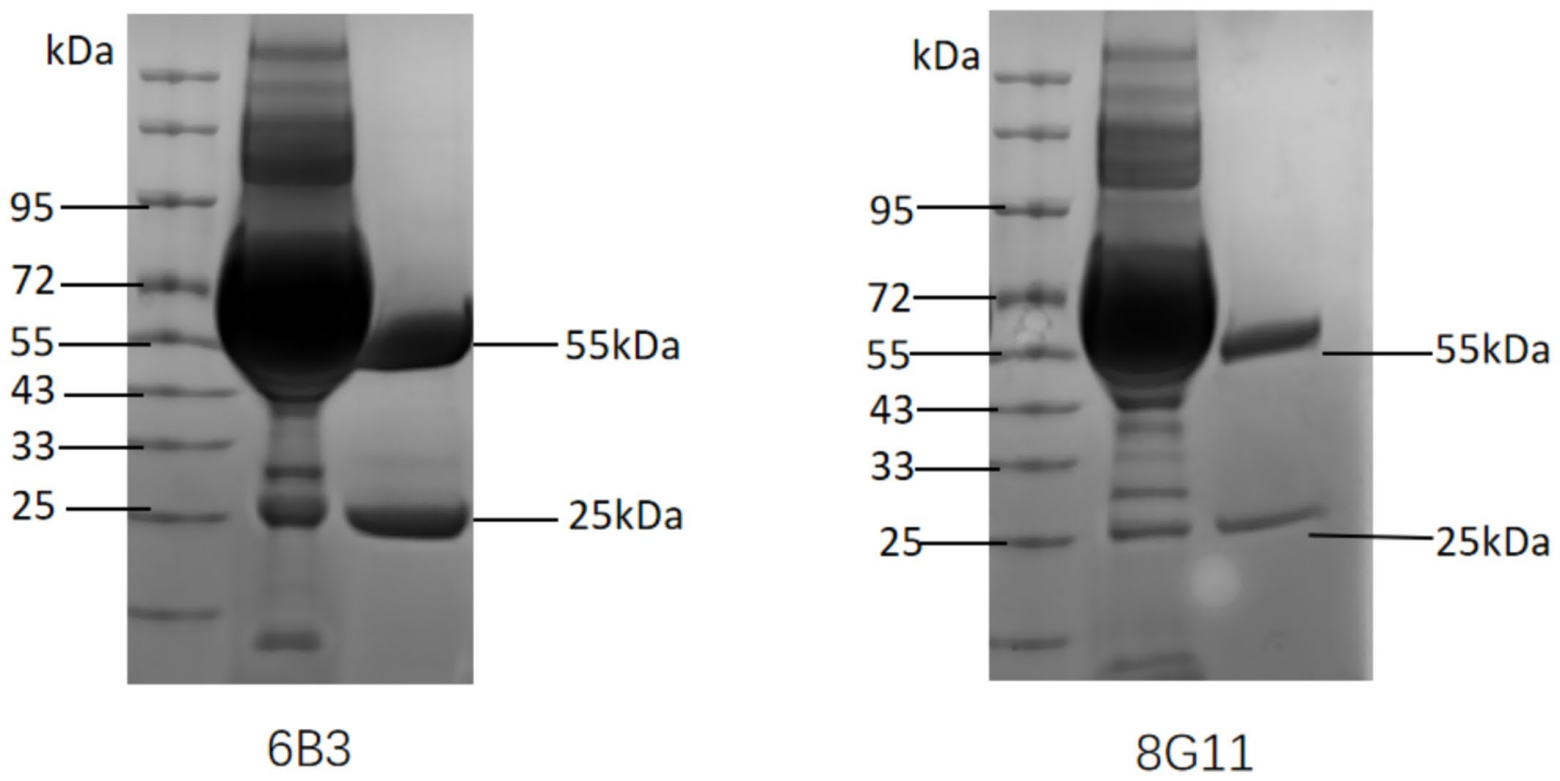
SDS–PAGE analysis of monoclonal antibodies. M: Protein marker; 1: before purification; 2: after purification.

In this study, two monoclonal antibodies (6B3 and 8G11) against the FAdV-4 penton protein were successfully revived and prepared. Indirect ELISAs demonstrated that both ascites antibodies exhibited titers of 1:100,000, indicating high affinity. SDS–PAGE analysis confirmed that after purification via protein A/G affinity chromatography, the ascitic fluid produced two distinct bands by SDS–PAGE (Figure 1): one heavy chain band at approximately 55 kDa and one light chain band at approximately 25 kDa. Following purification, the protein concentrations of the monoclonal antibodies 6B3 and 8G11 were determined to be 1.27 mg/mL and 0.55 mg/mL, respectively.

**Figure 2:**
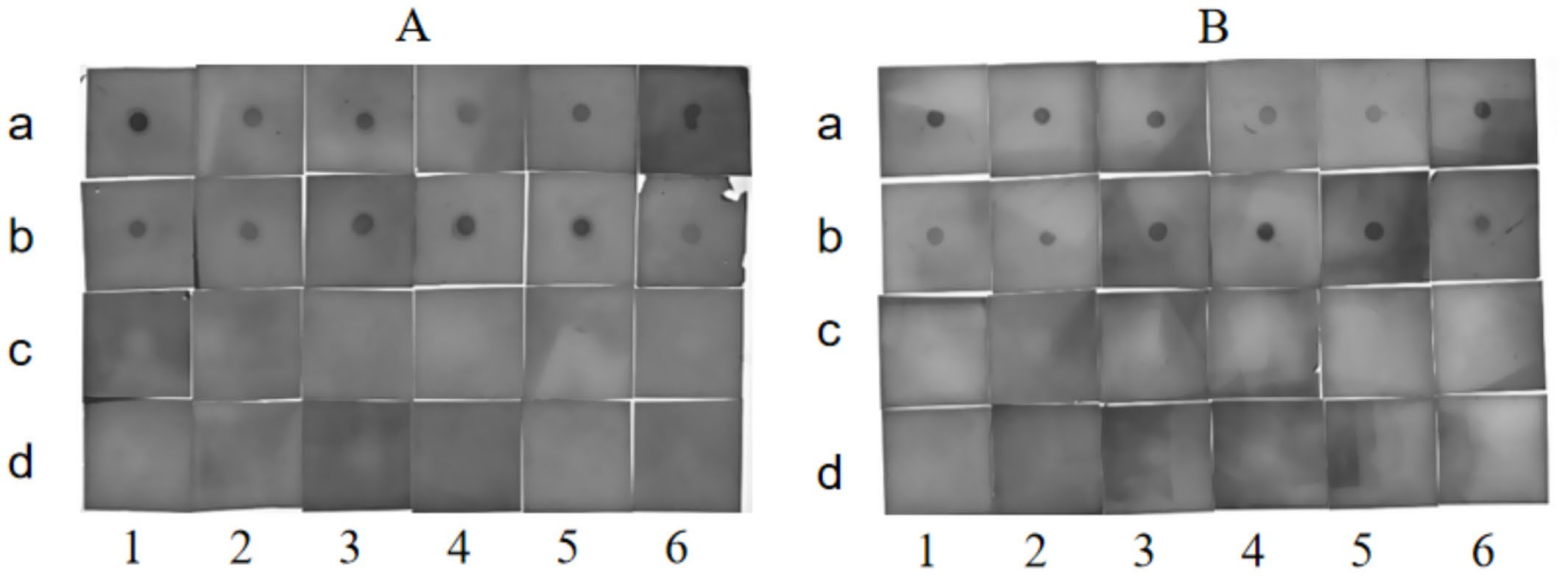
Dot-ELISA analysis of the monoclonal antibodies 6B3 **(A)** and 8G11 **(B)** against viral antigens. (a1): FAdV4-001, (a2): FAdV4-003, (a3): FAdV4-005, (a4): FAdV4-006, (a5): FAdV4-007, (a6): FAdV4-008, (b1): FAdV4-009, (b2): FAdV4-010, (b3): FAdV4-012, (b4): FAdV4-013, (b5): FAdV4-015, (b6): FAdV4-017, (c1): FAdV2, (c2): FAdV5, (c3): FAdV6, (c4): FAdV7, (c5): FAdV11, (c6): FAdV12, (d1): AIV H9, (d2): NDV, (d3): IBV, (d4): ILTV, (d5): EDSV, (d6): ARV.

The Dot-ELISA results demonstrated that both monoclonal antibodies 6B3 (Figure 2A) and 8G11 (Figure 2B) strongly bound to all 12 FAdV-4 isolates, as evidenced by distinct dark spot signals. Critically, no cross-reactivity was observed with other FAdV serotypes (FAdV-2, FAdV-5, FAdV-6, FAdV-7, FAdV-11 and FAdV-12) or another avian viruses (AIV H9, NDV, IBV, ILTV, EDSV and ARV), confirming exceptional epitope specificity. These findings validate that 6B3 and 8G11 are ideal diagnostic antibodies for broad-spectrum FAdV-4 detection, combining high sensitivity with unmatched serotype discrimination.

**Figure 3:**
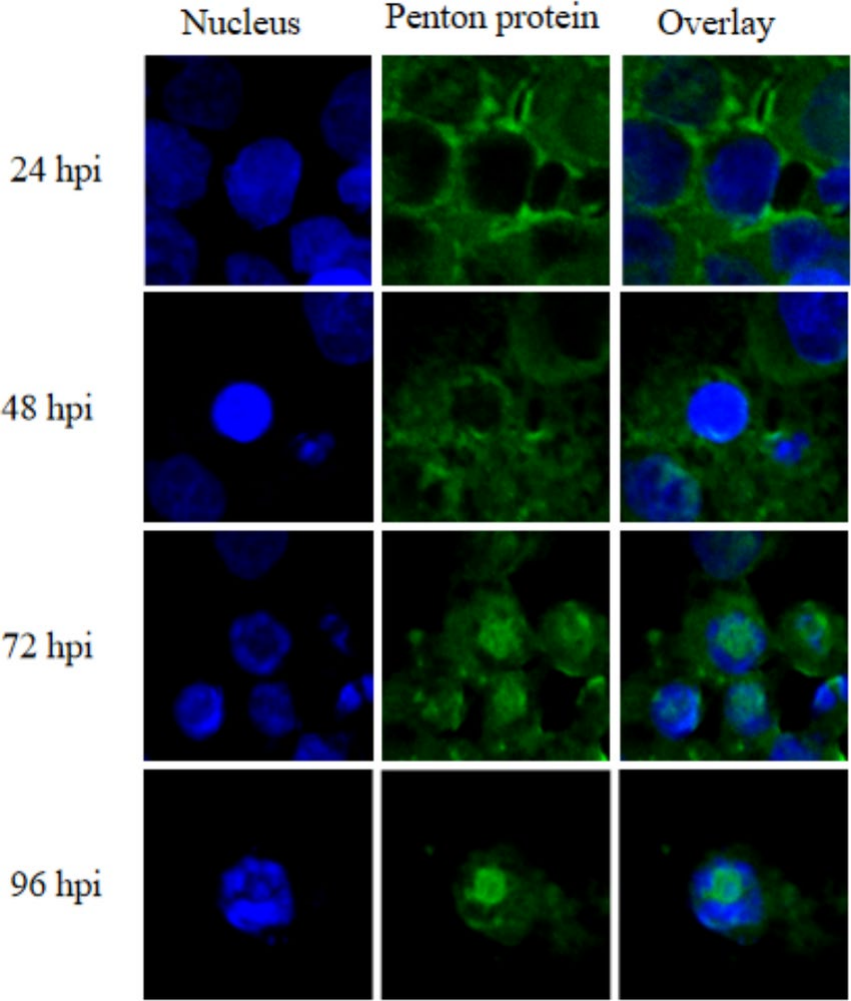
Subcellular localization dynamics of the penton protein following FAdV-4 infection in LMH cells.

Next, the spatiotemporal distribution pattern of the FAdV-4 penton protein during viral infection was systematically characterized using immunofluorescence microscopy with the specific monoclonal antibody 6B3. The results revealed a distinct phase-dependent localization pattern: during early infection (24 hpi), the protein primarily exhibited a diffuse cytoplasmic distribution with notable accumulation at the nuclear membrane periphery; at mid-infection stages (48–72 hpi), characteristic perinuclear aggregation foci formed while discrete intranuclear signals began emerging; and by late infection (96 hpi), complete nuclear localization was observed with perfect colocalization with DAPI-stained regions(Figure 3). This sequential “cytoplasmic→ perinuclear→intranuclear” translocation cascade demonstrates remarkable temporal–spatial consistency with the established biological process of viral capsid assembly, in which structural protein such as penton are progressively recruited to nuclear viral factories for virion morphogenesis.

### 3.2 Optimization of colloidal gold test strip

**Figure 4:**
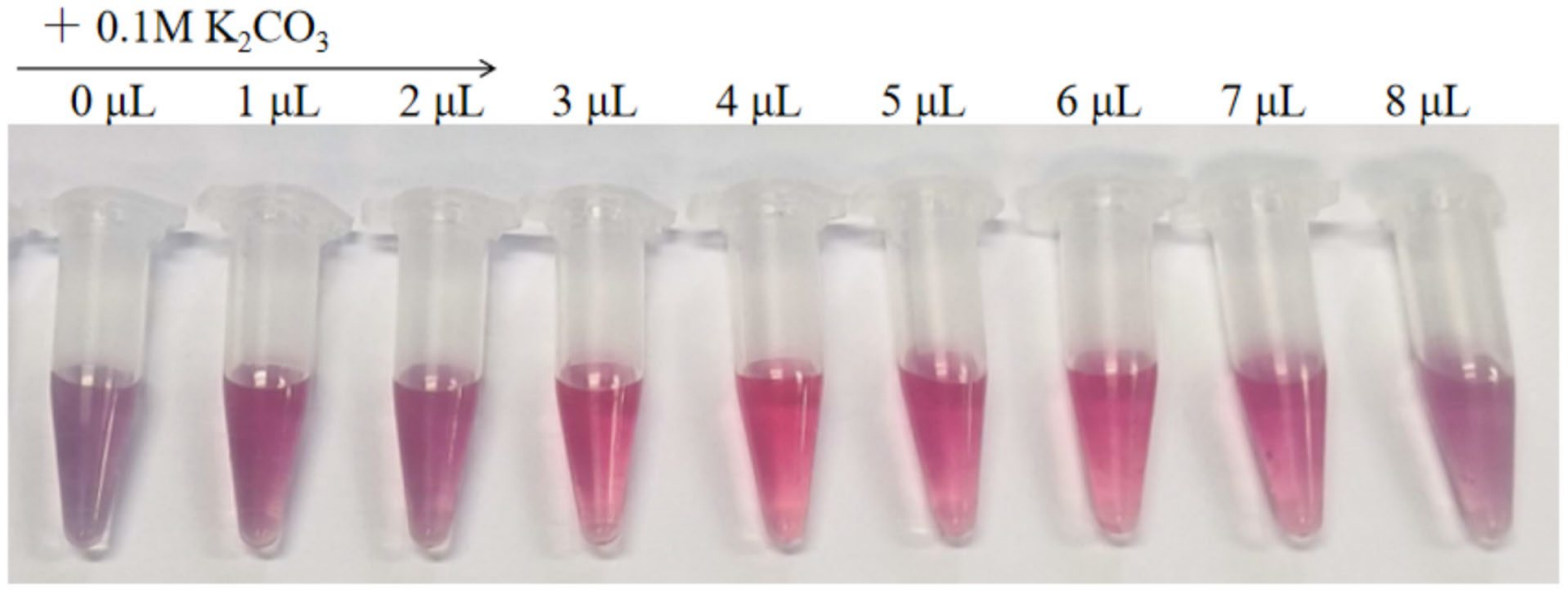
Results of the pH optimization for colloidal gold labeling.

Through pH optimization for colloidal gold labeling, the binding efficiency of the 6B3 monoclonal antibody to 40 nm colloidal gold under different pH conditions was evaluated. The experiment was set up with nine gradients (0–8 μL of 0.1 M K₂CO₃), and a clear color gradient was observed (Figure 4) from a purplish-red color on the left (0 μL, no alkali added) to a light pink color on the right (8 μL). When 3–4 μL of K₂CO₃ was added, the solution exhibited a stable wine-red color; under these pH conditions, the color remained stable after the addition of 10% NaCl, with no significant fading or aggregation. In contrast, samples with excessively low pH (0–2 μL groups) immediately turned bluish-gray after NaCl addition, indicating that ineffective antibody labeling led to colloidal gold aggregation. Moreover, samples with excessively high pH (5–7 μL groups) were light pink in color, suggesting that excessive antibody binding may have affected the labeling efficiency. On the basis of color stability, the addition of 4 μL of K₂CO₃ (pH ≈ 8.3) was determined as the optimal condition for 6B3 monoclonal antibody labeling. These results align with the classical pH range for colloidal gold labeling (pH 8.0–9.0), providing a critical parameter for the subsequent preparation of immunochromatographic test strips.

**Figure 5:**
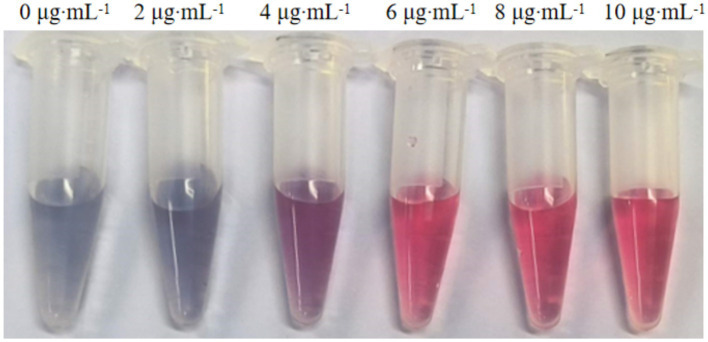
Optimization results for the colloidal gold-labeled antibody concentration.

Under the optimal pH condition, we optimized the dose of the labeled antibody. As shown in Figure 5, with increasing antibody concentration (0–10 μg·mL^−1^), the color of the colloidal gold solution changed substantially from the initial clear blue (0 μg·mL^−1^) to a stable wine red (≥6 μg·mL^−1^). The experimental results indicated that the minimum antibody concentration required to maintain the stability of colloidal gold was 6 μg·mL^−1^. To ensure the reliability and stability of the experiment, we ultimately selected 120% of this concentration (i.e., 7.2 μg·mL^−1^) as the optimal labeling concentration for subsequent experiments. This concentration not only ensures sufficient stability of the colloidal gold but also provides an appropriate safety margin to avoid potential stability issues.

**Figure 6:**
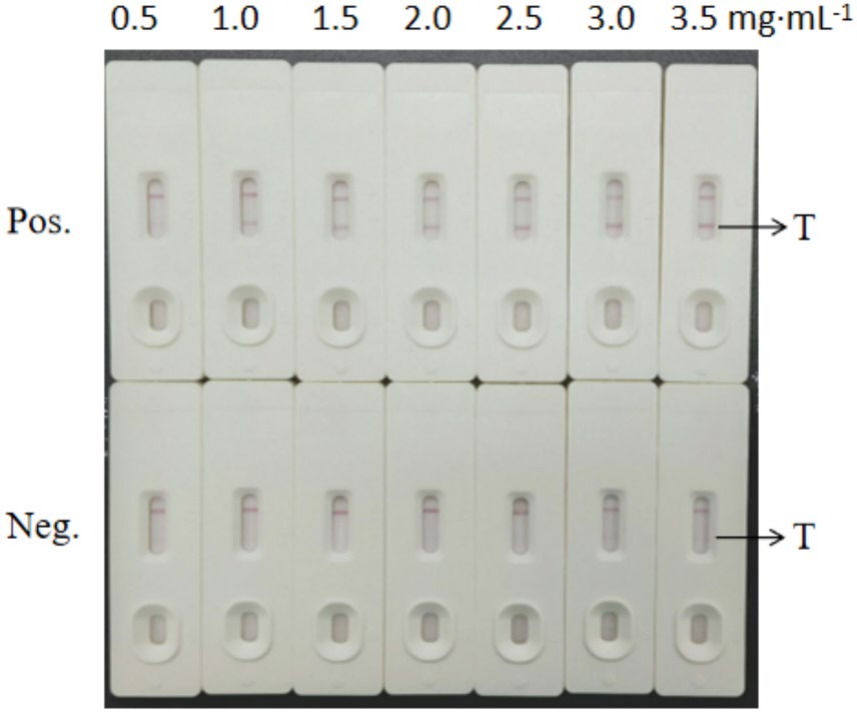
Screening test results for the T-line concentrations of the colloidal gold test strips.

As shown in Figure 6, we evaluated the effects of different antibody concentrations (0.5–3.5 mg·mL^−1^) on test line (T line) coloration using immunochromatographic test strips. The results from the positive group (Pos.) showed that as the T line antibody concentration increased from 0.5 mg·mL^−1^ to 2.5 mg·mL^−1^, the T line color gradually increased, and band clarity significantly improved. When the concentration reached 2.5 mg·mL^−1^, the T line exhibited a distinct band with optimal coloration. Further increasing the concentration to 3.0 mg·mL^−1^ and 3.5 mg·mL^−1^ did not significantly increase the color intensity or clarity of the T line, indicating that antibody binding had reached saturation. The negative group (Neg.) showed no visible bands at any concentration, confirming the absence of cross-reactivity and good specificity of the detection system. Considering both coloration effectiveness and cost efficiency, we selected 2.5 mg·mL^−1^ as the optimal working concentration for the T line antibody.

**Figure 7:**
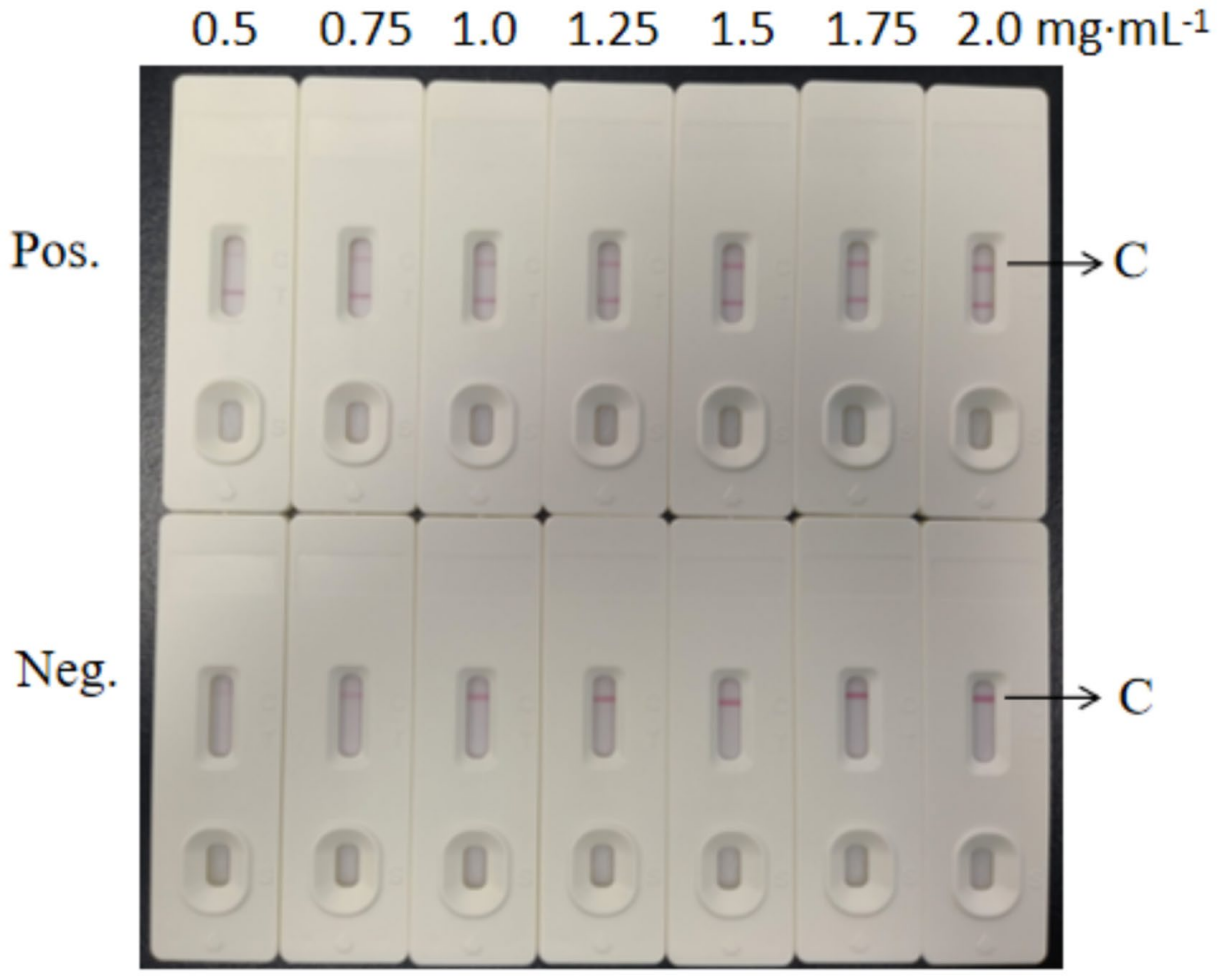
Screening test results for the C-line concentration of the colloidal gold test strips.

As shown in Figure 7, the evaluation of the effects of different antibody concentrations (0.5–2.0 mg·mL^−1^) on the coloration of the control line (C line) of the immunochromatographic test strip revealed the following results: When the C line antibody concentration was lower than 1.5 mg·mL^−1^, the positive group (Pos.) showed weak coloration (almost no color at 0.5 mg·mL^−1^ and light pink at 0.75 mg·mL^−1^). When the concentration reached 1.5 mg·mL^−1^, the C line exhibited a clear and stable red band, and further increasing the concentration to 2.0 mg·mL^−1^ did not significantly increase the color intensity. The negative group (Neg.) showed no bands at any concentration, indicating good specificity of the system. Considering both coloration effectiveness and cost efficiency, 1.5 mg·mL^−1^ was ultimately selected as the optimal spraying concentration for the C line. This concentration ensures detection sensitivity while avoiding antibody waste.

### 3.3 Performance evaluation of colloidal gold test strips

#### 3.3.1 Results of the specificity test

**Figure 8:**
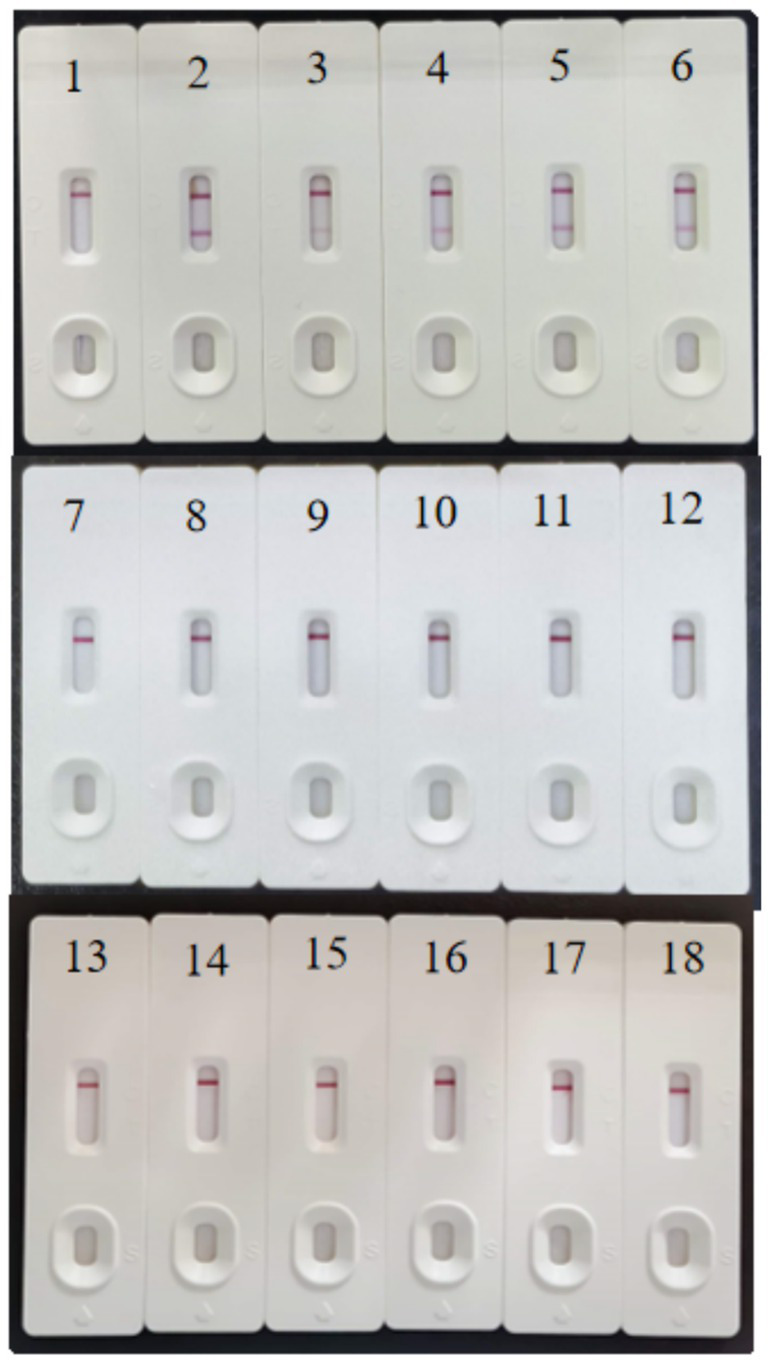
Results of the specificity test on the colloidal gold strips. 1: Negative control, 2: FAdV4-001, 3: FAdV4-005, 4: FAdV4-006, 5: FAdV4-007, 6: FAdV4-009, 7: FAdV2, 8: FAdV5, 9: FAdV6, 10: FAdV7, 11: FAdV11, 12: FAdV12, 13: AIV H9, 14: NDV, 15: IBV, 16: ILTV, 17: EDSV, 18: ARV.

A specificity evaluation of the test strips (Figure 8) revealed that sample 1 (negative control) presented only a C line, whereas samples 2–6 (FAdV4-GX001/005/006/007/009) presented clear dual T and C line bands, confirming the positive detection of FAdV4. In contrast, samples 7–12 (FAdV-2/5/6/7/11/12) and 13–18 (AIV H9, NDV, IBV, ILTV, EDSV and ARV) displayed only C lines without T line, indicating no cross-reactivity with other FAdV serotypes or common avian pathogens. These results collectively validate that the detection system specifically identifies FAdV4 while maintaining excellent specificity against related pathogens.

#### 3.3.2 Results of the sensitivity test

**Figure 9:**
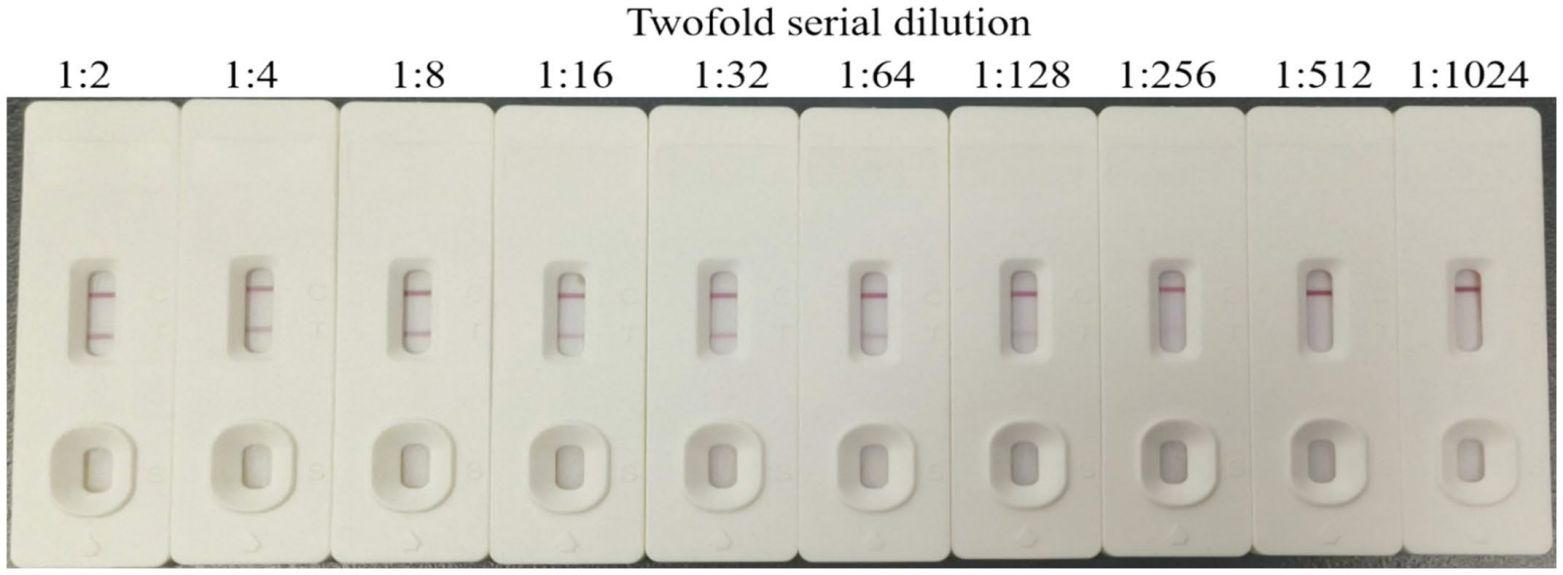
Sensitivity of the colloidal gold test strips.

The sensitivity test results are shown in Figure 9. After a 1:128 serial dilution of FAdV4-positive samples with a titer of 10^7^ TCID_50_/0.1 mL, the prepared test strips still yielded positive detection results. The calculated detection sensitivity of the test strips for FAdV4 was 7.81 × 10^4^ TCID_50_/0.1 mL.

#### 3.3.3 Results of the repeatability test

**Figure 10:**
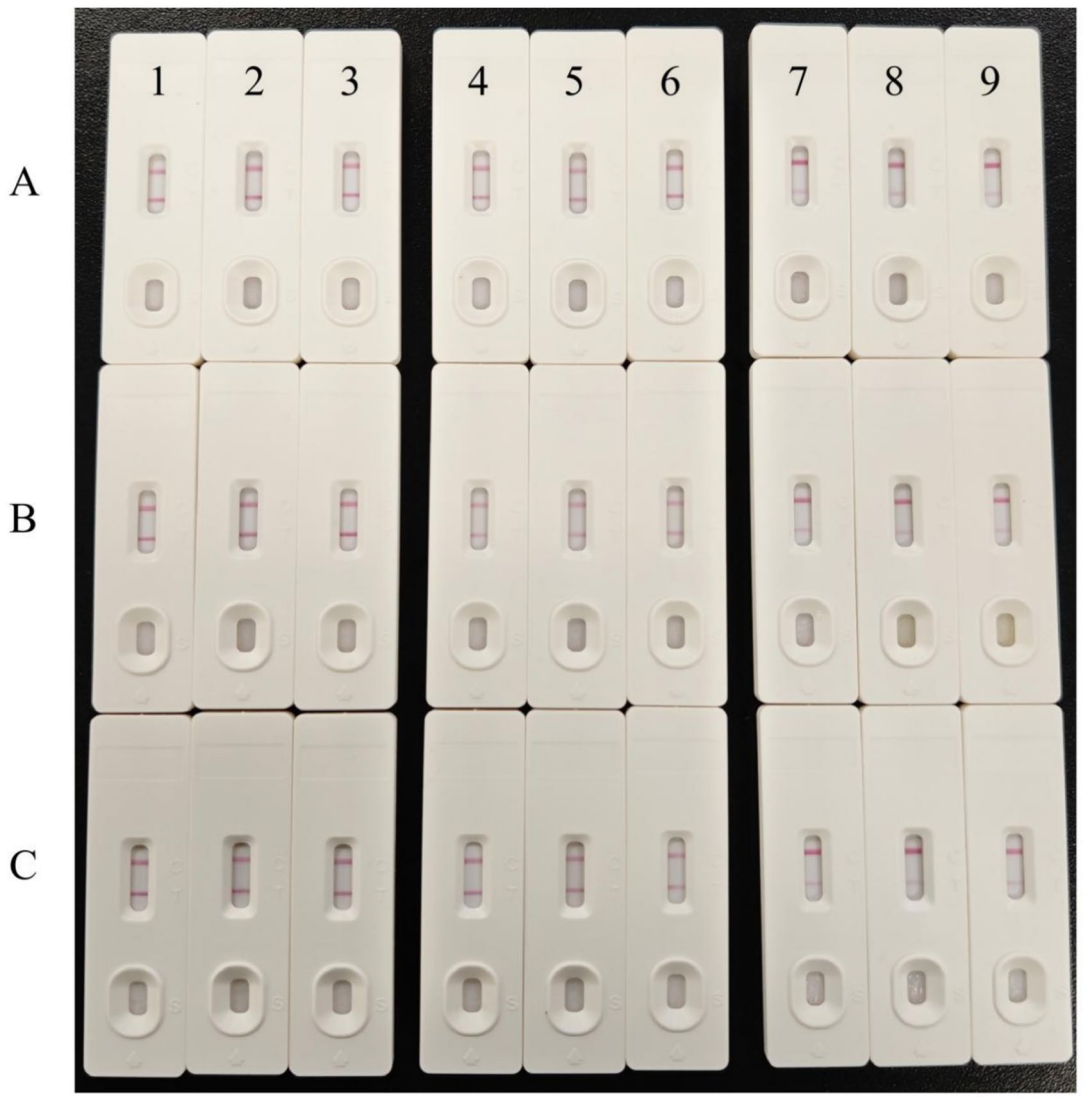
Repeatability of the colloidal gold test strips. 1–3: Repeatability for the first sample using test strips; 4–6: Repeatability for the second sample using test strips; 7–9: Repeatability for the third sample using test strips. **(A–C)** Test strips from different batches.

The repeatability test results of the colloidal gold test strip revealed that when the same batch of test strips was used to detect three randomly selected samples, with each sample tested three times, the results were completely consistent (Figure 10, 1–9), indicating excellent intra-batch repeatability. Furthermore, when three batches (Figures 10A–C) of test strips prepared at different times were used to test the three samples, the results from all the batches were also entirely consistent, demonstrating good inter-batch repeatability.

#### 3.3.4 Results of the stability test

The stability test results indicated that when the test strips were stored for 1, 2 and 3 months, the detection results for three randomly selected positive samples and three negative samples remained consistent. These findings confirm that the test strips exhibit excellent stability, with a shelf life of at least 3 months.

#### 3.3.5 Detection in clinical samples

A total of 152 cloacal and oral swab samples were collected from a poultry farm in Guangxi. The samples were detected using the developed colloidal gold immunochromatographic strip in this study and an ELISA method established in our laboratory (27). The results are shown in Table 1. Among the 24 ELISA-positive samples, 22 tested positive and 2 tested negative when the prepared test strips were used, yielding a positive agreement rate of 91.6% (22/24). All 128 ELISA-negative samples were consistently negative with the test strips, achieving a negative agreement rate of 100%. The overall agreement rate was 98.6% (150/152).

**Table 1:** The comparison of the detection results of both methods on the clinical samples.

Detection Methods	Positive	Negative	Total	Positive for both methods	Negative for both methods	Overall agreement rate
Colloidal gold test strips	22	130	152	22	128	98.6%(22+128/152)
ELISA	24	128	152			

## 4 Discussion

The 6B3 and 8G11 monoclonal antibodies successfully developed in this study demonstrated exceptional subtype specificity and broad-spectrum recognition capabilities. The Dot-ELISA results clearly indicated that both antibodies specifically recognized 12 different FAdV-4 isolates of various origins but showed no cross-reactivity with other FAdV serotypes or common avian pathogens. This high specificity stems from conserved antigenic epitopes on the surface of the penton protein, providing a molecular basis for establishing accurate diagnostic methods (28–31). Notably, the ascites antibody titer reached a high level of 1:100,000, and SDS–PAGE analysis revealed a typical IgG structure, laying a solid foundation for the subsequent development of detection technology.

Using immunofluorescence techniques, the spatiotemporal distribution characteristics of the penton protein during infection were systematically revealed for the first time. The observed “cytoplasmic → perinuclear → intranuclear” cascade transport pattern highly aligns with the biological process of viral capsid assembly (32). This discovery not only deepens the understanding of the FAdV-4 replication mechanism but also, more importantly, provides a theoretical basis for selecting the optimal diagnostic time window. Experimental data revealed that 48–72 hpi (hours post-infection) is the optimal detection window, during which viral protein expression is high and the distribution characteristics are distinct. These findings hold significant guiding value for improving the detection rate of clinical samples.

Through systematic optimization of the colloidal gold labeling parameters and detection line concentrations, a high-performance rapid test strip was successfully developed. The test strip is not only easy to operate and provides intuitive results but also exhibits excellent intra−/inter-batch repeatability and stability, making it particularly suitable for primary farms and veterinary clinical settings. The results are obtained within 15 min to significantly improve detection efficiency for the rapid diagnosis, and the detection meets clinical and onsite detection needs. Compared with traditional virus isolation (5–7 days) and PCR detection (18, 33), the test strip substantially reduces time costs and technical barriers, filling a technological gap in the on-site rapid detection of FAdV4.

The colloidal gold test strip developed in this study offers the advantages of rapid detection, operation simplicity, low cost, and making it well suited for onsite testing. However, the method of colloidal gold test strip still has some methodological limitations. First, its detection sensitivity, which relies on visual interpretation, is sufficient for most of the clinical onsite screening needs, but is lower than that of PCR and ELISA methods. Thus, it is more suitable for preliminary screening in cases with relatively high viral loads in the samples, or the false-negative results may be occurred as the samples from the early infection or when the viral load is too low, as that showed negative results in the two ELISA-positive samples in this study. Second, the method has certain sample matrix requirements: relatively clear samples such as oropharyngeal swab, urine and serum can be tested directly, whereas complex or viscous samples (e.g., tissue homogenates) require standardized pretreatment to avoid nonspecific interference. Third, the test strip provides qualitative or semiquantitative results as positive/negative or strong/weak positive, and cannot achieve precise viral load quantification. Although the test strips have the aforementioned limitations, owing to its ability to perform rapid onsite testing and the unique advantage of requiring no laboratory instrument for detection, it is an indispensable diagnostic preliminary screening tool.

## 5 Conclusion

In this study, a colloidal gold immunochromatographic test strip based on prepared anti-FAdV-4 penton protein monoclonal antibodies (6B3 and 8G11) was successfully developed, which demonstrated significant technical advantages over existing methods and substantial application value. The success of this study provide a rapid tool for diagnosing FAdV4 infection and conducting epidemiological investigations, which are important for the comprehensive prevention and control of HHS and the healthy development of the poultry industry.

## Data Availability

The original contributions presented in the study are included in the article/supplementary material, further inquiries can be directed to the corresponding author.
